# Detection of Quorum-Sensing Molecules for Pathogenic Molecules Using Cell-Based and Cell-Free Biosensors

**DOI:** 10.3390/antibiotics9050259

**Published:** 2020-05-16

**Authors:** Craig Miller, Jordon Gilmore

**Affiliations:** Bioengineering Department, Clemson University, Clemson, SC 29632, USA; clm6@g.clemson.edu

**Keywords:** quorum sensing, infection, biosensor

## Abstract

Since the discovery and subsequent use of penicillin, antibiotics have been used to treat most bacterial infections in the U.S. Over time, the repeated prescription of many antibiotics has given rise to many antibiotic-resistant microbes. A bacterial strain becomes resistant by horizontal gene transfer, where surviving microbes acquire genetic material or DNA fragments from adjacent bacteria that encode for resistance. In order to avoid significant bacterial resistance, novel and target therapeutics are needed. Further advancement of diagnostic technologies could be used to develop novel treatment strategies. The use of biosensors to detect quorum-sensing signaling molecules has the potential to provide timely diagnostic information toward mitigating the multidrug-resistant bacteria epidemic. Resistance and pathogenesis are controlled by quorum-sensing (QS) circuits. QS systems secrete or passively release signaling molecules when the bacterial concentration reaches a certain threshold. Signaling molecules give an early indication of virulence. Detection of these compounds in vitro or in vivo can be used to identify the onset of infection. Whole-cell and cell-free biosensors have been developed to detect quorum-sensing signaling molecules. This review will give an overview of quorum networks in the most common pathogens found in chronic and acute infections. Additionally, the current state of research surrounding the detection of quorum-sensing molecules will be reviewed. Followed by a discussion of future works toward the advancement of technologies to quantify quorum signaling molecules in chronic and acute infections.

## 1. Introduction

**Figure 1:**
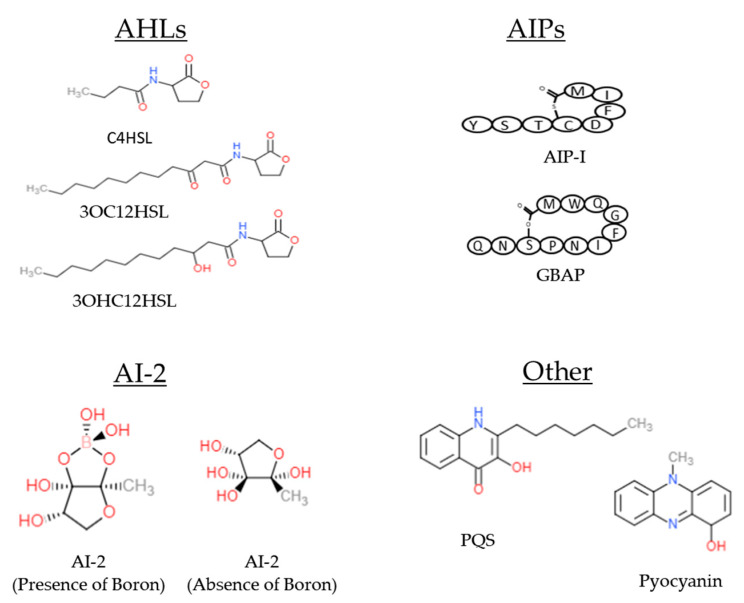
Chemical structure of common autoinducers discussed in this review.

Antibiotic resistance is a growing public health concern that threatens the effective treatment of infectious diseases. According to the Centers for Disease Control and Prevention (CDC), multidrug-resistant (MDR) bacteria infect at least 2.8 million people, and cause over 35,000 deaths per year [1]. Both Gram-positive and Gram-negative strains utilize a complex quorum-sensing (QS) communication system to regulate gene expression. Bacterial quorum-sensing networks control biofilm formation, virulence factor production, bioluminescence, and antibiotic resistance [2]. Quorum sensing depends on specific signaling molecules, called autoinducers, which accumulate in the extracellular space once the bacterial concentration reaches a specific threshold. Bacteria passively release or actively secrete low molecular weight acyl-homoserine lactones (HSLs) or autoinducing peptides (AIPs), respectively. AIPs are modified oligopeptides that can range from 5 to 17 amino acids [2]. HSLs are neutral lipid molecules that contain a lactone ring with varying carbon side chains. The extent of hydrophobicity depends on the length of the side chains. Autoinducer-2 (AI-2) is an interspecies signaling molecule used in the formation and progression of biofilms with multicellular bacterial communities [2]. AI-2 is a furanosyl borate diester used by several Gram-positive and Gram-negative species [3]. The pseudomonas quinolone signal (PQS) is a separate class of autoinducer used specifically by *Pseudomonas aeruginosa* (*P. aeruginosa*) to regulate virulence. Figure 1 shows the chemical structure of some common autoinducing molecules. After a critical threshold has been reached, autoinducers bind to transcription factors in the cytoplasm or cell membrane, activate gene expression, and produce more signaling molecules [4,5]. Rapid detection of these signaling molecules could give clinicians an early indication of infection. Conventional detection methods used to quantify and identify autoinducers include the use of mass spectrometry (MS) and/or high-performance liquid chromatography (HPLC). These systems are used to examine the physical/chemical properties of autoinducers [6]. MS and HPLC detection systems are highly accurate in the detection of autoinducers and are also used to validate novel biosensing schemes. Commonly used biosensor systems for in vitro and in vivo applications include the use of plasmids, chromosomes, or enzymes from reporter bacteria strains to detect a colorimetric, luminescent, or fluorescent signal [7]. QS biosensors have the potential to detect bacterial virulence prior to the expression of resistant genes. Antibiotics could then be given at an optimal time to maintain antibiotic stewardship. Furthermore, QS biosensors could be used to screen for novel anti-virulent, antimicrobial, and/or quorum quenching molecules. Thus, further advancement and validation of these biosensors for in vitro and in vivo virulence detection could lessen the MDR bacteria epidemic. 

## 2. Development of Antibiotic Resistance in ESKAPE Bacteria

The development of multidrug-resistant ESKAPE strains could be attributed to the overuse of antibiotics. Broad-spectrum antibiotics are often prescribed as the first line of defense for wounds suspected of having significant bacterial colonization. However, when bacteria are in advanced stages of biofilm formation and virulence, governed by QS, these bacteria enable mechanisms for resistance to antibiotic treatment which begins the pathway for multidrug resistance. Administration of antibiotics during the stationary phase could also produce persister cells and future resistance. According to a study done with the IMS Health MIDAS database, there has been a 36% increase in antibiotic drug use between 2000 and 2010 [8]. Moreover, approximately 80% of antibiotics in the U.S. are used in the agriculture industry in order to increase yield, quality, and profit. The use of antibiotics in crops and livestock can promote infections in humans and transfer resistant genes to host pathogens [9]. According to the World Health Organization, there has been a decrease in the development of approved antibiotics over the past few decades [10]. Antibiotic approvals by the FDA decreased from 16 between 1983 and 1987 down to two between 2008 and 2012. Although this number has increased to seven between 2013 and 2017, the Infectious Diseases Society of America believes that more novel drugs are needed to reverse the MDR pandemic [11].

Most antibiotics work by destroying the bacteria cell wall, preventing DNA replication, inhibiting protein synthesis, and/or by depriving the cell of vital nutrients. Bacteria can also be intrinsically resistant or acquire specific antimicrobial resistance genes via horizontal gene transfer. For example, Gram-positive pathogens are intrinsically resistant to aztreonam while Gram-negative pathogens are resistant to glycopeptides and lipopeptides. From a molecular standpoint, bacteria resistance could be due to drug inactivation, target modification, activation of drug efflux, and/or decreased drug uptake. Target pathogens can make surface modifications in order to prevent antibiotic attachment. Methicillin-resistant *Staphylococcus aureus* (*S. aureus*) prevents the binding of penicillin by synthesizing the penicillin-binding protein 2a through the *mecA* gene which binds to any drug with a β-lactam group [12,13]. Bacteria inactivate drugs by complete degradation or modification of a chemical group. Penicillin resistance in *S. aureus* is due to the synthesis of a β-lactamase called penicillinase. Hydrolyzation of the amide bond in penicillin and ampicillin inactivates the drugs [12]. Overexpressed efflux pumps remove toxic compounds which would prevent the proper accumulation of antibiotics to kill the cell. Overexpression of the *S. aureus* NorA efflux pump can lead to resistance of tetracycline. [12,13].

**Figure 2:**
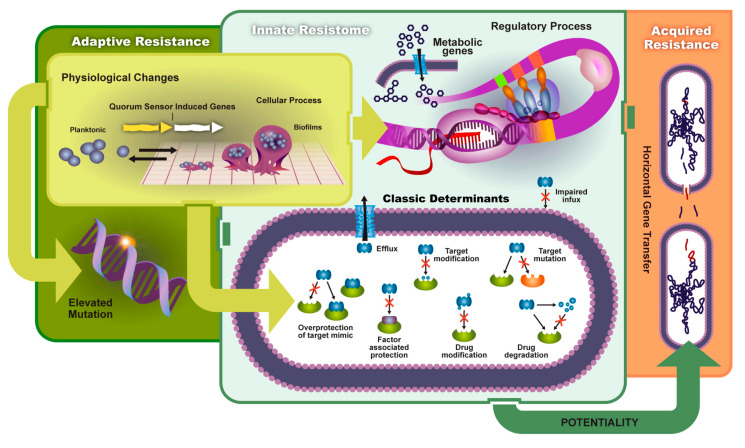
Pathways to antibiotic resistance via biofilm formation and quorum-sensing (QS) regulated gene transfer or innate resistance. Antibiotic resistance is caused by target mutation, drug efflux activation, drug modification, and uptake reduction. Reprinted with permission from [16]. Copyright 2017 MDPI.

Biofilms contribute to the reduction of drug uptake and the formation of adaptive (environmental) resistance. Bacterial biofilm formation begins in the planktonic state where cells are motile until they attach to an adequate surface and bind with other cells. This initial adhesion state is weak, but further progression leads to the formation of an extracellular matrix composed of extracellular DNA, exopolysaccharides, and other proteins. Figure 2 shows a schematic of biofilm formation and antibiotic-resistant pathways discussed in this section. QS plays a vital role in the production of the extracellular polymeric substance (EPS) and the release of virulent genes. The EPS enhances cell–cell communication and increases horizontal gene transfer. Pathogens contained in a mature biofilm structure are 1000 times more resistant than planktonic cells due to this increased QS efficiency. Persister cells, slow growth of bacteria, and poor antibiotic penetration decrease antimicrobial efficacy. Thus, higher concentration dosages are needed to reduce infection [14,15]. 

## 3. QS in Gram-Positive Pathogens

Gram-positive bacteria utilize AIPs to regulate QS networks. These AIPs are first produced in the cytoplasm of the bacterial cell. Then they are actively secreted from the cytoplasm by specific AIP transporters located in the cell membrane. Once the pathogens reach a concentration threshold in the extracellular environment, AIPs are detected by membrane-bound two-component sensor kinase receptors, which autophosporylates at histidines located in the cytoplasm. The interaction between AIPs and the sensor kinase receptors begins the activation of the respective quorum systems [4,17]. Table 1 summarizes the QS networks discussed in this section. 

*S. aureus* is a commensal microbe and human pathogen that has the potential to cause a wide range of infections. It is a key contributor to bacteremia, endocarditis, skin/soft tissue, and device-related infections. The accessory gene regulator (Agr) is the main QS system of *S. aureus* [18]. The Agr operon activates many toxins and degradative enzymes [19,20,21,22]. P2 and P3 promoters activate the RNAII and RNAIII divergent transcripts, respectively. P2 promoter activation results in the expression of *agrA*, *agrB*, *agrC*, and *agrD* genes. The *agrD* and *agrB* transcripts are responsible for the production and secretion of AIPs, respectively. The *agrD* gene encodes a precursor molecule and synthesizes extracellular QS AIPs. AgrB is needed to actively secrete autoinducers through the cell membrane via transmembrane proteins. A two-component signal transduction system is encoded by the *agrC* and *agrA* genes. AgrC histidine kinase sensor becomes phosphorylated once it binds to an AIP. AgrA is the response regulator to the *agrB* gene, which binds to the P2, P3, PSMα, and PSMβ peptide promoters leading to the production of toxins, surface proteins, and exoenzymes [20,21]. 

*Enterococcus faecium* (*E. faecium*) and *Enterococcus faecalis* (*E. faecalis*) are commensal organisms found in the gastrointestinal tract that have the potential to become virulent and cause nosocomial infections [23]. Similar to the Agr system, these bacteria primarily use the Fsr system to regulate its quorum-sensing dependent virulence factors. This system contains four major genes: *fsrA*, *fsrB*, *fsrC*, and *fsrD*. The *fsrD* and *fsrB* produce and secrete gelatinase biosynthesis-activating pheromone (GBAP), respectively. FsrC is the sensor histidine kinase that becomes phosphorylated after binding to GBAP, which activates the FsrA response regulator [24]. The activation of FsrA upregulates the expression of *fsrBCD*, *gelEsprE*, and *ef1097* [25,26,27]. 

*Streptococcus pneumoniae* (*S. pneumoniae*) causes bloodstream infections, pneumonia, meningitis, and acute otitis media [28]. These bacteria utilize two QS networks: Com and the Lux/AI-2. The Com pathway utilizes the competence-stimulating peptide (CSP) signaling molecule where its precursor is synthesized by the *comc* gene, converted to CSP by the ComAB transporter, and detected by the membrane-bound histidine kinase, ComD. The Lux/AI-2 system is like the system discussed above. Both systems are important in biofilm regulation [29,30].

## 4. QS in Gram-Negative Pathogens

Gram-negative bacteria use HSLs as signaling molecules to regulate gene expression in a concentration-dependent manner. However, some strains produce additional non-homoserine lactone molecules, such as the PQS in *P. aeruginosa* [31,32]. Most Gram-negative systems contain a LuxI/LuxR QS network homolog. LuxI is responsible for the synthesis of the HSLs that are passively diffused through the cell membrane. At high cell density levels, HSL ligands bind to highly specific LuxR-type transcriptional factors. Conversely, HSLs are degraded at a rapid rate at low cell density levels to avoid premature virulence activation. Binding of LuxR-type factors allows for the activation of target virulence genes, auxiliary QS networks, and the production of additional HSLs. HSL side carbon chains can range from short to long, where they can be 4 to 16 carbon chains long [4].

The opportunistic pathogen, *P. aeruginosa*, causes acute and chronic nosocomial infections in immunocompromised individuals. Infections are commonly found in burn victims and patients with cystic fibrosis [33,34]. *P. aeruginosa* consists of three major QS networks that function in a hierarchical manner. Upon activation of the Las system, the remaining networks (rhl and pqs) are positively regulated. Rhl is also upregulated by the pqs system. The compounds used to signal the activation of each system are *N*-(3-oxododecanoyl)-l-homoserine lactone (3-oxo-C12-HSL), *N*-butanoyl-l-homoserine lactone (C4-HSL), and 2-heptyl-3-hydroxi-4-quinolone for the las, rhl, and pqs systems, respectively. The las and rhl systems are of the LuxR/LuxI type network as discussed in the above section [35,36]. 

*A. baumannii* is responsible for nosocomial infections to include pneumonia, endocarditis, skin, and wound infections. It uses acyl-homoserine lactones ranging from 10 to 16 acyl side carbon chains. These lactones are medium- to long-chain HSLs [37,38]. Most strains produce and detect 3-hydroxy-C12-homoserine lactone (3-oxo-C12-HSL) to regulate its QS circuit. Pathogenic *A. baumannii* strain M2 produces 3-oxo-C12-HSL and utilizes this lactone as its main signaling molecule. The QS circuit contains AbaI and AbaR that is like the LuxI and LuxR system found in *P. aeruginosa*. AbaI produces the HSL signaling molecules and AbaR is the corresponding receptor protein that is responsible for the expression of target QS genes [37]. 

*Klebsiella pneumoniae* (*K. pneumoniae*) causes nosocomial, urinary tract, and surgical wound infections [39,40]. The QS system in *K. pneumoniae* is less clear than the other ESKAPE pathogens. However, there is evidence that it utilizes type-two autoinducers or 4,5-dihydroxy-2,3-pentanedione (AI-2) and HSLs in its QS circuit [39,40,41]. A report showed that *K. pneumoniae* isolates from a human tongue produced *N*-octanoylhomoserine lactone and *N*-3-dodecanoyl-l-homoserine lactone [41]. AI-2 is produced by a LuxS system, which is an enzyme found in several bacterial species. Thus, Al-2 is involved in interspecies QS communication and found in both Gram-negative and Gram-positive bacteria [42,43]. There is little known about the transcriptional factors involved in the production of QS signaling molecules. Thus, this would be a great opportunity for further research. 

*Enterobacter* spp. QS network: *Enterobacter* spp. is a family of Gram-negative bacteria associated with nosocomial infections that has the ability to cause meningitis, septicemia, wound infection, and other complications [44]. There is little evidence about specific transcriptional factors involved in its virulence production. However, Zhou et al. and Yin et al. were able to isolate long- and short-chain HSLs, respectively [45,46]. This is an indication that *E.* spp. has the ability to synthesize and secrete HSLs using a LuxR-type QS network. [46] Gram-negative bacteria *Escherichia coli* (*E. coli*), *Salmonella typhimurium* (*S. typhimurium*), and *Vibrio harveyi* (*V. harveyi*) all have a LuxS/AI-2 system that utilizes type-2 autoinducer. Many strains of *E. coli* and *V. harveyi* are used in the construction of biosensors to detect type-2 QS systems in other strains [47].

## 5. State-of-the-Art Biosensing for Quorum-Sensing Molecules

Conventional methods to verify the presence of QS signaling molecules and biosensor functionality include the use of chromatography, mass spectrometry, or a combination of the two. High-performance liquid chromatography-tandem mass spectrometry (HPLC-MS/MS) procedure is mostly used to detect acyl-homoserine lactones, peptides, and other biologically relevant compounds [50,51]. HPLC-MS/MS combines the separation capabilities of HPLC and the detection technique of MS. Briefly, the analyte is passed through a chromatographic column and separated by HPLC then the components are transferred to a mass spectrometer where samples are converted to positively charged particles bombarded by electrons. Charged particles are detected by a mass spectrometer and their mass-to-charge ratio is calculated. HPLC-MS/MS requires the use of ion detectors, mass analyzers, an ion source, a vacuum system, and other highly expensive equipment [52,53]. HPLC-MS/MS is great for verifying the presence of quorum-sensing signaling molecules and validating biosensor functionality. Many researchers utilize HPLC-MS/MS to detect short-, medium-, and long-chain homoserine lactones and autoinducing peptides secreted in bacterial cultures [6,51,54,55,56,57]. 

## 6. Biosensing Developments

Conventional screening methods provide a way to identify pathogens and diagnose most infections. However, their use can be time-consuming and economically challenging in certain situations. The utilization of biosensors has several advantages over conventional methods. Most biosensors used to detect QS activity are cheap and easy to use. Point-of-care along with telemedicine can be used to diagnose chronic or acute infections in economically challenging areas. Sensors have been made to determine the bacterial load in vitro [58,59,60]. However, these sensors are susceptible to common biological interferences. Additionally, biosensors were developed that utilize antibodies or bacteriophages to specifically detect bacteria [61,62,63]. The incorporation of antibodies and/or bacteriophages solves the interference problem, but it does not guarantee that the captured bacteria are pathogenic. It was widely accepted that a bacterial load above 10^6^ CFU/mL indicates the onset of infection [64]. However, many researchers believe that the release of virulence factors and biofilm production hinders the healing process and serves as a better indication of infection [64,65,66]. Bacterial colonization is only the presence of bacteria and the use of antibiotics before a “critically colonized” level could lead to antimicrobial resistance [67]. Virulent and/or critically colonized bacteria can be verified by the detection of toxins or QS signaling molecules. Genetically modified whole-cell-based bacteria, cell-free transcriptional-based reagents, aptasensors, and immunosensors are commonly used to detect these compounds in vitro or in vivo. 

### 6.1. Gram-Positive

*Gram-Positive Whole-Cell-Based QS biosensing*: Autoinducing peptides (AIPs) are great targets for quorum-sensing activity detection in Gram-positive bacteria. Peptide-based biosensors have been used to target various molecules to include proteins, enzymes, and nucleic acids by exploiting the selectivity of synthetic and natural peptides as enzymatic substrates. The cleavage between the analyte and peptide is verified through bio-conjugation with signal markers to produce a quantifiable output signal [68]. However, most AIP biosensing applications utilize a variety of engineered plasmids that have cognate AIP receptors and promoters that produce a measurable readout. Transcriptional-based biosensing allows researchers to detect AIPs with high specificity and sensitivity. Thus, genetically engineered bacteria are the gold standard when detecting AIPs [69]. Several plasmids have been developed to detect CSP (*Streptococcus* spp. signaling molecules), synthesized by *S. pneumoniae*. A lacZ transcriptional reporter strain (*S. mutans* SMdC) was developed to assess the activity of comDE in response to CSP. The strain consisted of a *comC* mutant that prevents the production of CSP. A promoterless *lacZ* gene was used to fuse vectors pYH2 and pOMZ47 to the *comDE* and *nlmAB* promoters, respectively. Exogenous CSP induced the expression of Beta-galactosidase (β-gal) in both mutans. Quorum sensing activity was assessed by the activation of β-gal as a percentage of maximal activation. A detection range was not determined from this study [70,71]. 

A group from Norway developed two biosensors to quantify gelatinase biosynthesis-activating pheromone (GBAP) and cytolysin small subunit (CylL_S_) [72]. The CylL_S_ biosensor was constructed by electroporation of the pSL101cylR2R1Pcyl vector into *E. faecalis* JH2-2. The GBAP biosensor was developed from the use of two vectors: pREG696*lux*P_fsr_B45 and pREG696*lux*P_gelE_. Bioluminescence was measured in CylL_S_ inducing units (CIU) and GBAP inducing units (GIU). The authors found that the P_gelE_-driven *lux*ABCDE had a greater *lux* expression than the P_fsr_ promoter. The vector containing the P_gelE_ promoter was transformed into *E. faecalis* V583fsrB. Both biosensors were tested in *E. faecalis* cell-free supernatants that produced CylL_S_ and GBAP. The GBAP sensor displayed a bioluminescence equivalent to 320 and 5120 GIU in exponential phase and overnight cultures, respectively. This showed that GBAP activity was not responsible for the downregulation of the fsr circuit in the stationary growth phase. CylL_S_ biosensor was also able to detect CylL_S_ in cell-free supernatants during the exponential (640 CIU) and stationary (5120 CIU) growth phase [72]. 

**Figure 3:**
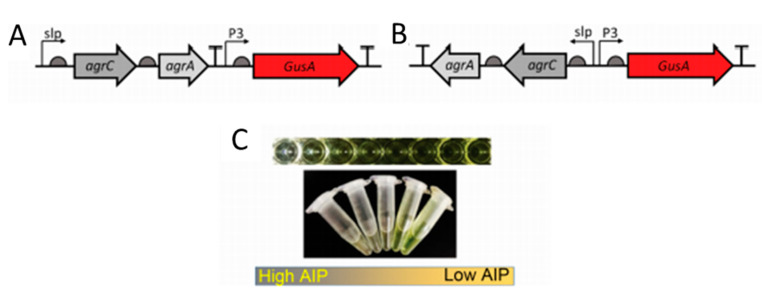
Probiotic biosensor schematic. (**A**) Sensor 1 with pSIP409 plasmid. (**B**) Sensor 2 with pSIP409 plasmid and a flipped *slp*-*agrC* fusion. (**C**) GusA reduction in the presence of high autoinducing peptides (AIPs). Reprinted with permission from [73]. Copyright 2018 American Chemical Society.

Most cell-based biosensors consist of non-pathogenic mutant bacteria derived from pathogenic strains. However, there still exists a risk when using such bacteria. Lubkowicz et al. reprogrammed the probiotic bacteria, *Lactobacillus reuteri* DSM20016, to detect AIP-I from *S. aureus*. The group designed two different reporter bacteria. Gibson assembly was used to construct all plasmids. The *agrCA* genes and p3 promoter were amplified from the *S. aureus* RN 4220 strain. Plasmid pSIP409 was used to amplify the reporter GusA and can be seen in Figure 3. The second sensor consisted of the same sequence with a flipped slp-AgrCA site from the first designed plasmid. *E. coli* Top 10 was used to make plasmid copies and plasmids were transformed to *L. reuteri* via electroporation. The sensors innately produce glucuronidase. Therefore, the authors used p-nitrophenyl β-D-glucuronide (pNPGA), which hydrolyzes in the presence of glucuronic acid. Sensor 1 was inversely proportional to AIP-I with a detection range between 10 and 1000 nM. However, Sensor 2 was developed to address small inadvertent expression of GusA from Sensor 1 when incubated with *S. aureus* cell lysate. Sensor 2 addressed a leaky GusA expression, had the same inverse response to AIP-I, but with a lower detection range of 0.5–1000 nM [73].

A variety of 11 different plasmids were created via the amplification of PCR promoters and subsequent vector ligation by Malone et al. to detect *agr* expression. Plasmids developed by Yarwood et al. were used as base vectors for the construction of all 11 plasmids [74,75]. The *asp23* promoter and a YFP reporter gene were amplified from *S. aureus* SH1000 and pDB59 plasmid as templates, respectively. The resulting PCR products were processed and ligated into pDB59 to create the first plasmid, pAH5. Plasmids pAH6 and pAH1 were constructed with an *agr23* and *agr* P3 promoter, respectively, that expressed mCherry. The remaining plasmids were created with different promoters downstream of a gene encoding various fluorescent proteins. Table 2 annotates all plasmids constructed in this study. The authors found that the ribosome binding site (RBS) played a role in protein expression. Due to the higher expression of protein (YFP), superoxide dismutase (*sod*) RBS and delta-toxin (*hld*) RBS, plasmids pAH16 (sod RBS), and pAH17 (hld RBS) were superior out of all plasmids [74,76]. Application of plasmids in *S. aureus* biofilm growth and fluorescence-activated cell sorting was also validated [74]. Two plasmids were generated to respond to exogenous S. aureus peptides. *E. coli* was used as a vehicle for plasmid construction and finalized plasmids were electroporated into *S. aureus* MN8. Plasmid pDB60 was transformed into an MN8 to construct an *agrD* mutant with *ermC* (erythromycin resistance cassette), *agrAC*, and *agrB* P3. Plasmid pDB22 was constructed and contained a fusion of the agr P3 promoter downstream of a gene encoding GFP. A second plasmid, pJY202 contained a fusion of the agr P2-P3 promoter in line with a gene encoding YFP. Average fluorescence was four times and 14 times higher than the required fluorescent signal needed for visualization. In a liquid media culture, both plasmids displayed an increase in fluorescence signal from late exponential to stationary growth phase. The group successfully examined biofilm formation under flow cytometry and what influence the Agr system had on antibiotic resistance [75]. Boles et al. constructed a plasmid, pAH9, with a *sarA* P1 promoter downstream from an *mCherry* gene using PCR amplification. The plasmid was subsequently transformed into S. Aureus SH1000. The researchers were able to use the reporter strain to show that low *agr* activity increased biofilm formation and AIPP-I causes biofilm detachment. Planktonic and dispersed cells showed an increased sensitivity to the antibiotic, rifampicin [74]. 

### 6.2. Gram-Negative

*Gram-Negative Whole-Cell-Based QS biosensing: Agrobacterium tumefaciens* (*A. tumefaciens*) is a Gram-negative bacterium that infects plants by the growth of tumors via horizontal gene transfer. The bacteria, however, are not pathogenic to humans. *A. tumefaciens* utilize a TraI/TraR-type quorum-sensing circuit to regulate its virulence in plant species through the introduction of a tumor-inducing plasmid. Tumor-inducing plasmids maintain a copy of the transfer DNA and regulate its conjugation. TraI is the synthase that synthesizes 3-oxo-C8-HSL and TraR is its transcriptional activator [77]. In order to detect HSLs, especially 3-oxo-C8-HSL, the synthase must be deleted or mutated. *A. tumefaciens* NT1 and NTL4 are commonly used as a host for several HSL biosensors with a broad detection range and can screen for medium- and long-chain HSLs. NT1 and NTL4 do not produce HSLs due to a mutation of the TraI synthase or complete removal of the tumor-inducing plasmid. An NT1 strain that consists of a pZLR4 plasmid was used to detect several 3-oxo acyl-HSLs and 3-hydroxyl acyl-HSLs with different carbon side chains. The pZLR4 plasmid has *traR* and a *traG::lacZ* reporter. This plasmid allows for resistance to ampicillin and gentamicin. In the presence of X-gal, the reporter strain produces β-galactosidase that then provides a blue color indicating the presence of HSLs listed in Table 3 [80,81]. pZLR4 plasmid has been widely used by several researchers in NT1 and NTL4 strains [78,80,81,82,83]. *A. tumefaciens* A136 has been used to quantify several HSLs. Chambers et al. and Zhu et al. used pCF218-pMV26 and pCF218-pCF372 to screen and quantify HSLs, respectively [79,84]. Plasmid pMV26 has a PtraI promoter fused to reporter *luxCDABE* reporter. While plasmid pCF372 contains a PtraI promoter fused with a reporter *lacZ* gene. TLC bioassay reporter was used to detect the presence of 3OC10HSL, 3OC8HSL, and 3OC12HSL from the sputum samples of cystic fibrosis patients with the pCF372 plasmid [84]. Luminescence-based assay was used to quantify HSLs with various side carbon chains: C4HSL (25 nM), C6HSL (250 nM), C8HSL (0.25 nM), C10HSL, (25 nM), C12HSL (250 nM), 3OC6HSL, (20 pM), 3OC8HSL (0.2 pM), 3OC10HSL (0.02 pM), and 3OC12HSL (0.02 pM) [84]. Zhu et al. used *A. tumefaciens* to detect and quantify a broad range of HSLs using the TLC bioassay and β-galactosidase activity. Bacteriophage T7 was used to engineer a TraR protein expression system in *A. tumefaciens* KYC55 using three plasmids: pJZ384, pJZ410, and pJZ372. Plasmid pJZ384 has a *traR* gene with a phage T7 promoter, pJZ410 has the phage *T7 RNA polymerase* gene and the last plasmid has a *traI* gene fused to reporter *lacZ*. Biosensor was able to detect 3 pM of 3OC8HSL by detecting the amount of β-galactosidase activity. TLC bioassay was used to detect several HSLs: 3OC6HSL (2.5 pM), 3OC8HSL (0.25 pM), 3OC12HSL (0.5 nM), 3hydroxylC6HSL (20 pM), 3hydroxylC8HSL (20 pM), 3hydroxylC6HSL (20 pM), C6HSL (100 pM), C8HSL (30 pM), and C10HSL (40 pM). The biosensor has greater sensitivity and a lower detection limit than other *A. tumefaciens* biosensors [85].

**Figure 4:**
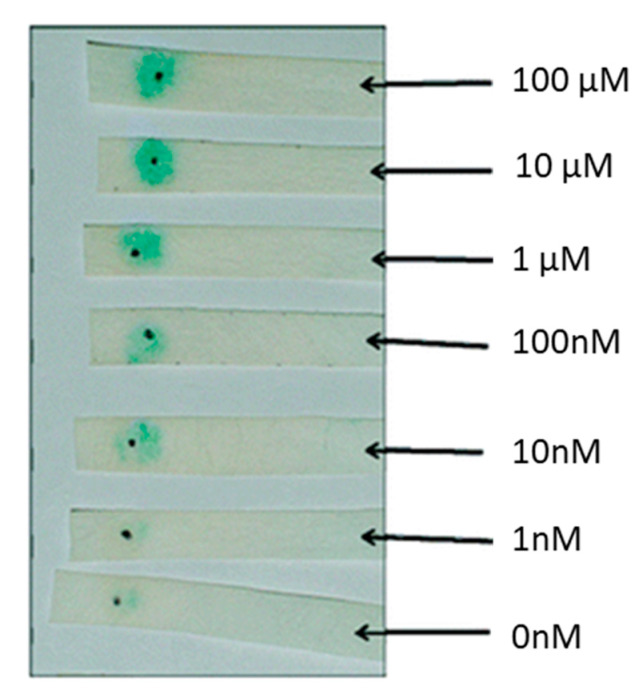
C12HSL *E. coli* DH5α-T1 paper biosensor. Reprinted with permission from [93]. Copyright 2010 American Chemical Society.

*E. coli* is widely used in detecting QS signaling molecules and its proteins and plasmids are used to derive cell-free lysates. Conveniently, *E. coli* is inherently absent of an HSL synthase gene. Most *E. coli*-based biosensors use a *lux*CDABE or β-galactosidase reporter system. Several biosensors based on a *lux*CDABE or *lacZ* reporter system have been developed in the latter half of the 20th Century [86,87,88]. Since the last two decades, many authors utilize *E. coli*-based plasmid systems. Recently a bioluminescent biosensor developed by Winson et al. was used to quantify HSLs. This sensor uses the JM109 *E. coli* strain as a host and harbors a pSB1075 plasmid. The psB1075 plasmid has a LasR receptor capable of activating a lux reporter in response to 3OC12HSL and other HSLs. As expected, there was a 10-fold signal reduction for every two-carbon side chain (C12HSL, C14HSL, and C16HSL) when compared to 3OC12HSL. The biosensor had a 10-fold increase in response to 10 nM of 3OC14HSL and 3OC16HSL than the cognate 3OC12HSL. The biosensor was able to detect 12 different non-cognate HSLs tested except for C18HSL [89]. Rai et al. designed a fluorescent biosensor capable of detecting synthetic HSLs. *E. coli* K-12-Z1 strain was used as a host to carry the pSB1A2 plasmid. The biosensor was able to detect 1 µM of synthetic HSLs and was able to validate a computational model capable of showing that promoter logic can be used to probe and predict QS activity [90]. Deng et al. found a way to quantify 3OC6HSL with a visible cherry fluorescence and determine biosensor location with green fluorescence. The dual fluorescent whole-cell-based sensor contains the plasmid pUCGMA2T_1-4_, which has three components: PnptII fused to gfp to indicate host biosensor, PahlI fused to *mcherry* to quantify 3OC6HSL, and the *ahlR* gene to encode HSL regulatory protein. The biosensor was able to detect 5 × 10^−8^–1 × 10^−5^ mol/L of HSL [91]. HSL concentrations were detected in real-time using *E. coli* fluorescent reporter bacteria. A plasmid encoding a gfpmut3 signal in the presence of HSLs was used to detect fluctuations of HSLs in real-time. *E. coli* MT102 cells containing the pJBA132 plasmid were able to detect the presence of 10 nM of 3OC6HSL in approximately 15 min via an epifluorescence microscope. The gfp signal had a 40 min half-life, which allows this biosensor to be used in real-time detection schemes. Fluorescence in response to HSL dose was measured in liquid cultures at different time points to determine the detection limit of various HSLs. The detection limits were 1 nM, 10 nM, 10 nM, 10 nM and 1000 nM for 3OC6HSL, C6HSL, C8HSL, 3OC10HSL, and C4HSL, respectively [92]. A portable system was designed to detect C12HSL using a paper strip whole-cell-based sensing. As can be seen in Figure 4, the biosensor can be used in point-of-care applications to quantify infection. The pSDB1075 plasmid has a *lacZ* reporter that expresses β-galactosidase under the control of a *lasI* promoter. The plasmid was transformed into competent *E. coli* DH5α-T1. Reporter cells and chromogenic substrate X-gal (5-bromo-4-chloro-3-indolyl-β-d-galactopyranoside) were immobilized on filter paper via physical adsorption. The limit of detection was 10 nM of C12HSL for 90 min and 100 nM after 60 min of incubation [93].

Several *Pseudomonas*-based biosensors have been constructed that utilizes a *lux*CDABE or *lacZ* reporter. *Pseudomonas* spp. Produce HSLs, so most sensors consist of plasmids with a mutating *lasI* or *rhII* HSL synthase gene. Massai et al. created a chromosomally integrated whole-cell biosensor that only bio-illuminates in the presence of 3OC12HSL and C4HSL. The sensor has a *lasI* mutant in the *P. aeruginosa* PA14 strain with a chromosomal copy of a P*rsaL* promoter fused to a *lux*iCDABE reporter. The detection range was 1.4 nM to 3 µM with a lower limit of detection for 3OC12HSL of 10 pM. The sensor responded to C4HSL with a lower detection limit of 10 µM [86]. Dong et al. utilized a *Pseudomonas*-based whole-cell biosensor to show that *PprB* expression is essential for virulence production. *P. aeruginosa* mutant strain M71LZ was used to assess the production of β-galactosidase in the presence of HSLS. The strain contains a plasmid with *lacZ* fused to a *rsaL* promoter. The detection range was between 0.01 and 100 µM and 1.0 and 100 µM for 3OC12HSL and C4HSL, respectively. Additionally, the authors were able to show that a mutation in the *pprB* gene reduced gene expression (*lasI*, rhII, and *rhIR*) and 3C12HSL production. However, the *pprB* knockout strain was sensitive to C4HSL. This study shows that PprB upregulates the production of 3OC12HSL in *P. aeruginosa* PAO1 [94]. *P. fluorescens* is a bacterial species commonly cultured from human clinical samples and found in soil samples. There is a disparity in the literature as to whether *P. fluorescens* is pathogenic or not [95]. Khan et al. developed a whole-cell-based biosensor to detect HSLs in *P. fluorescens* 2–79 culture supernatants. The *phzR* gene in the *P. fluorescens* genome can detect 3OC6HSL and the *phzA* gene upregulates the production of phenazines (redox-active metabolites). Production of phenazines gives their host a survival advantage in polymicrobial environments and has beneficial properties towards plants. Two plasmids (pSF105 and pSF107) were transformed in the *P. fluorescens* 1855. The *phzR* gene was cloned to a pSF105 plasmid. The pSF107 plasmid had a *phzR*-phzA dual promoter region. The *uidA* and *lacZ* reporters were placed upstream of the *phzR* and *phzA* promoter, respectively. The detection range of 3OC6HSL via β-galactosidase assay was between 10 and 10,000 nM. The biosensor was able to detect C6HSL and 3OC8HSL, but with a much lower sensitivity. The authors believe that the sensor is best for detecting 3OC6HSL. Additionally, it was noted that the 2–79 strain uses 3OC6HSL as its QS signal and PhzR activates *phzA* and *phzR* expression [96]. Interspecies crosstalk was analyzed using two different biosensors. Two different plasmids were constructed and transferred (triparental mating) to *B. cepacia* H111-1 and *P. aeruginosa* PAO1-JP2 cells to specifically detect 3OC12HSL (pKRC12) and C8HSL (pASC8). Plasmid pKRC12 contains components from the *P. aeruginosa* PAO1 las system, which has a lasB fused together with gfp and the *lasR* gene under control by a *lac* promoter. The second plasmid, pASC8, utilized components from the *B. cepacia* H111 cep QS network. The plasmid has a gfp under the control of a cepi promoter and a *cepR* gene transcribed by a *lac* promoter. The detection range for plasmids in *P. aeruginosa* was both 25 nM for their respective HSL. The group was able to show that interspecies communication is a one-way direction in *P. aeruginosa* PAO1 and *B. cepacia* H111 biofilm cultures. *B. cepacia* was able to recognize 3OC12HSL from *P. aeruginosa*, but the latter was unable to uptake HSLs from *B. cepacia* [97].

Reporter mammalian cells are of great interest. Mammalian cells such as fibroblasts are involved in the inflammatory response in chronic and acute infections. They are responsible for the destruction of damaged structural proteins and the creation of a new extracellular matrix [98]. Fibroblast-like, monkey kidney COS-1 cells, were used to develop an in vitro mammalian reporter cell-culture system to detect the presence of C4HSL and 3OC12HSL [99]. Reporter plasmids consisted of a LasBOX 1 sequence upstream of a *firefly luciferase* gene. Mammalian cells have different mechanisms to control transcriptional activity than that of prokaryotic cells. Therefore, the group incorporated a protein (T3N) with three copies of the VP16 transcriptional domain and the nuclear localization signal derived from an SV40 T antigen. Luciferase Assay Kit was used to detect luciferase activity in response to HSLS. Luciferase assay showed that reporter cells with a T3N module fused to RhIR had a three-fold signal increase vs. reporter cell with only an N module fused to RhIR in the presence of C4HSL (250 µM). T3N-LasR chimeric proteins were used to test time response to 3OC12HSL (250 µM) and C12HSL (750 µM) over a 48-h period. Although a higher concentration of C12HSL was needed for maximal effectiveness, both ligands induced a maximal response at 8 h with a significant drop off over the remaining 48-h. This inactivation could be due to HSL-inactivating enzymes or apoptosis of cells. This system could be used to assess mammalian cell response in the presence of active QS systems, screen for HSL mimics, or HSL inhibitors [99].

Whole-cell biosensing systems are very robust. However, cell-free systems also have their advantages. Cell-free systems allow the synthesis of proteins and contain the enzymes from eukaryotic or prokaryotic cells needed for transcription and translation [100]. Kawaguchi et al. utilized the *A. tumefaciens* NTL4 with pCF218 and pCF372 plasmids to make a cell-free assay system to detect QS HSLs. β-galactosidase activity was detected under 4 h in the presence of C6 HSL (30 nM), C7HSL (25 nM), C8HSL (20 nM), C10HSL (100 nM), C12HSL (200 nM), 3OC6HSL (17 nM), and 3OC8HSL (10 nM). However, the cell-free lysate was unable to detect C4HSL [101]. USER-ligase DNA templates were used with *E. coli* S30 to generate libraries of DNA components for cell-free systems. PCR products were prepared using *PfuTurbo* C_x_. PCR-amplified products were then circularized and used as DNA templates for cell-free systems via gfp production. All promoters and ribosome binding sites tested were able to express gfp in the presence of 3OC12HSL. Additionally, promoters and RBS had a similar response to HSLs in cell-free and cell-based extracts. However, two (J23100 and J23102) out of nine different promoter constructs had a significant increase in signal strength in the presence of 3OC12HSL. The group was also able to compare linear versus circular (plasmid) DNA cell-free systems to *E. coli*-based systems with the pSB1A2 plasmid. This in vitro approach allows one to rapidly investigate several different constructs for synthetic biology applications [102]. The pSB1A2 plasmid developed by Chappel et al. was used to detect 3OC12HSL concentration levels in cystic fibrosis patients. The detection range of the biosensor was between 5 and 100 nM. Twenty *P. aeruginosa* positive samples were tested for HSL with LasRV biosensor and LC-MS. Biosensor had one false negative and one false positive out of the 20 samples. The two discrepancies could be due to molecular suppression or activation from interferents in samples [103]. 

Colorimetric methods for the detection of a wide range of HSLs typically consist of a *Chromobacterium violaceum* mutant. *C. violaceum* is a Gram-negative soil and water bacterium that produces violacein through the CviI/R QS circuit. Mclean et al. designed a *C. violaceum* mutant (*C. violaceum* CV026) that is incapable of synthesizing HSLs. The thin-layer chromatography method is commonly used with this reporter strain. The biosensor is the most sensitive to C6HSL, but is able to detect C4HSL, C8HSL, 3OC6HSL, and 3OC8HSL [104]. Furanosyl borate diester (AI-2) is a signaling molecule used by both Gram-positive and Gram-negative bacteria to regulate QS activity [82]. *V. harveyi* BB170 reporter strains are commonly used to detect and quantify AI-2 molecules. This strain does not produce AI-2 due to a LuxS knockout nor sensitive to HSLs. The bioluminescence produced is due to AI-2 concentration and can be detected via the luminometer of the spectrometer [105]. 

*Gram-Negative Aptamer-Based QS biosensing*: There are limited developments in the use of aptamers to specifically detect QS signaling molecules or toxins produced by bacteria discussed in this review. Aptamer sensors include the use of fragmented DNA to detect cognate strands. Typically, a redox probe is used to detect the hybridization event between target and probe aptamer. Systematic evolution of ligands by exponential enrichment (SELEX) is a commonly used tool to develop highly specific aptamers for many applications. A group of aptamers was developed with a high affinity towards 3OC12HSL and C4HSL with a dissociation constant of 20 nM–35 nM and 25 nM–50 nM, respectively [106]. These aptamers could be used to quench or detect HSLs released during infection. Sismaet used aptamers developed by Zhao et al. to electrochemically detect 3OC12HSL and C4HSL. Methylene blue was used as a redox label, which was attached to the aptamer probe. Once cognate ssDNA binds to the aptamer probe, the distance between the redox label and electrode surface increases. Thus, the peak current at a −0.25 V is inversely proportional to the HSL concentration. The range of detection for 3OC12HSL and C4HSL was between 0.1 and 100 µM [107]. 

*Gram-Negative Electrochemical-Based QS biosensing*: An electrochemical sensor was developed to detect the presence of β-galactosidase produced by the *A. tumefaciens* NTL4 reporter. β-galactosidase hydrolyzes 4-aminophenyl β-D-galactopyranoside (PAPG) into p-aminophenol (PAP). PAP production was more sensitive than the detection of PAPG consumption. PAPG is irreversible, so any saturation in signal response is due to the complete consumption of PAPG. The concentration of 3OC12HSL was detected electrochemically *A. tumefaciens* 3OC12HSL spiked cultures. Limit of Detection in bacterial cultures was 2.5 and 3.6 pM in 2 h and 5 h of incubation, respectively [108]. Other HSLs could be detected with this method due to NTL4 response to HSLs outlined in Table 3. The Rhl and PQS quorum systems are responsible for the upregulation of pyocyanin via *phz*ABCDEFG operons. Pseudomonas Quinolone Signal (PQS) is the QS molecule in the PQS system. Pyocyanin is synthesized from the chorismate precursor, which is converted to phenazine-1-carboxylic acid (PCA). The PhzM and PhzS enzymes convert two other precursors to upregulate the production of pyocyanin. PQS has an oxidation and reduction peak at +0.233 V and +0.178 V, respectively. Pyocyanin can be detected via electrochemically with symmetrical peaks at −0.16 V and an irreversible oxidation peak at +0.8 V [32].

## 7. Outlook and Considerations 

Biosensing of QS molecules is a promising technique for the quantification of infection at the POC and has important implications to enhance antibiotic stewardship and reduction of multidrug-resistant strains. Further work is needed to fabricate QS biosensors that can rapidly and specifically detect the progression of antimicrobial resistance.

Autoinducers are critical to the pathogenicity and virulence of pathogens. Moreover, inhibition of these signaling molecules or mutations in regulator genes has been shown to disrupt virulence [109,110,111,112]. Early quantification of these molecules and toxins in vitro or in vivo would give an early indication of infection and facilitate the proper use of appropriate therapeutics. Whole-cell-based biosensors have been used to detect both autoinducing peptides and acyl-homoserine lactones. Moreover, in the past decade, more effort has been made to develop cell-free transcriptional biosensors to detect autoinducers. These systems have the advantage of detecting HSLs without the need of using cultured bacteria. However, most cell-free biosensors are developed to detect HSLs and there is little to no reports on the use of cell-free systems capable of detecting autoinducing peptides. Clinically relevant HSL concentrations found in cystic fibrosis sputum samples are in the 0.9 nM–6 µM range [113,114,115]. More research is needed to understand the concentration range of HSLs in various infected wound environments. There is also limited to no reports on the concentration of autoinducing peptides in wound cultures. Thus, clinically relevant concentrations of HSLs and AIPs in vivo would allow researchers to develop useful sensors that can be used to detect the onset of infection.

Most cell-based and cell-free systems are derived from pathogenic species. Although they are genetically modified to minimize virulence, they could induce an immune response by the host if used directly at an infection site. In order to develop in situ autoinducing whole-cell or cell-free systems, non-pathogenic bacteria should be used. Fluorescence is a commonly used reporter technique. An excitation source and a dark enclosure are needed to quantify fluorescence. Alternatively, other methods such as colorimetric, aptamers with electrochemistry, or odor emission reporter systems could be used for in situ applications. Interestingly, novel aptamers have been developed to specifically bind to autoinducing analogs. However, the number of reported aptamer-based sensors are extremely limited and further work could be done to synthesize more aptamers for QS biosensing applications. The detection of autoinducers produced by pathogenic bacteria would give clinicians information about the type and stage of infection, which would minimize the progression of antimicrobial resistance.

## Figures and Tables

**Table 1:** QS networks and key players in ESKAPE bacteria.

Bacteria Strain	QS System	Main Signaling Molecules	Transcriptional Factor	QS Virulence Regulation	Ref.
*S. aureus*	Agr	AIP-I,II,III	AgrA	lipases, proteases, enterotoxins, superantigens, ureases	[7,20,21,48]
	LuxS	AI-2	LuxR-type	capsular polysaccharide synthesis	
*E. faecium*/*E. faecalis*	Fsr	GBAP	FsrABCD	Cytolysin, gelatinase	[7]
*S. pneumoniae*	Com	CSP	ComE	polysaccharide capsule, pneumolysin	[7,29,30]
	LuxS	AI-2	LuxR-type	Biofilm formation	
*K. pneumonia* *e*	LuxS	AI-2, 3OC10HSL, C8HSL	LuxR	Antibiotic resistance genes, biofilm formation	[7,39,40,41]
*A. baumannii*	Aba	3OC12HSL, C12HSL, C10HSL, C14HSL, 3OC13HSL, C16HSL	AbaI/AbaR	Biofilms, siderophore, lipopolysaccharides, superoxide dismutase	[7,37,38]
*P. aeruginosa*	Las	3OC12HSL	LasR/LasI	Elastase (lasB), staphylolysin (lasA), alkaline protease (aprA), exotoxin A (toxA), hydrogen cyanide synthase (hcnABC)	[7,35,36]
	Rhl	C4HSL	RhlR/RhlI	Rhamnolipid synthase (rhlAB), type 1 lectin (lecA), type II lectin (lecB), hcnABC, pyocyanin	
	PQS	2-heptyl-3hydroxy-4-quinolone (PQS)	PqsR	Pyocyanin, lecA, rhlAB, lasB	
	LuxS	AI-2	LuxR-type	Biofilm formation	
*E. coli*	LuxS	AI-2	LsRB	Chemotaxis towards AI-2	[47,49]
*V. harveyi*	LuxS	AI-2	LuxP	bioluminescence	[7]
*E.* spp.	LuxR-type	C12HSL, short-chain (C6) HSL molecules	LuxR	Biofilm formation	[45,46]

**Table 2:** Gram-positive detection schemes.

Gram-Positive Detection Schemes				
Host Strain/Cell	Plasmid/Biorecognizing Element	Reporter System	Molecules	Detection Range/LOD
*S. pneumoniae* SMdC	pYH2-pOMZ47	LacZ reporter/β-gal	CSP	Not reported [70,71]
*E. faecalis* JH2-2	pSL101cylR2R1Pcyl	Bioluminescence	cytolysin	640 CIU [72]
*E. faecalis* MMH594	pREG696luxPfsrB45 and pREG696luxPgelE	Bioluminescence	GBAP	320 GIU [72]
*L. reuteri* DSM20016	pSIP409	GusA	AIP-I	10–1000 nM [77]
*L. reuteri* DSM20016	pSIP409 (w/flipped slp-AgrCA)	GusA	AIP-I	0.5–1000 nM [77]
*S. aureus* SH1000	pAH1 (*agr* P3)	YFP, Cam	*agr* expression *agr* expression	Not reported [78,79]
	pAH5 (SigB)	YFP, Cam		
	pAH6 (*asp23*)	mCherry, Cam		
	pAH7 (*agr* P3)	YFP, Erm		
	pAH8 (*agr* P3)	mCherry, Erm		
	pAH12 (*sarAP1*)	mCherry, Erm		
	pAH13 (tetracycline ind.)	GFP, Erm		
	pAH14 (*sarAP1*)	YFP, Erm		
	pAH15 (pAH14 w/*SarA* RBS)	YFP, Erm		
	pAH16 (pAH14 w/*sod* RBS)	YFP, Erm		
	pAH17 (pAH14 w/*hld* RBS)	YFP, Erm		
	pAH9 (*sarA* P1)	mCherry		

**Table 3:** Gram-negative detection schemes.

Gram-Negative Detection Schemes				
Host Strain/Cell	Plasmid/Biorecognizing Element	Reporter System	Molecules	Detection Range
*A. tumefaciens*	pZLR4	LacZ reporter/β-gal	C6HSL C8HSL C10HSL C12HSL C14HSL 3OC6HSL 3OC8HSL 3OC10HSL 3OC12HSL 3OHC8HSL 3OHC6HSL	Mostly quantitative [78,80,81,82,83]
*A. tumefaciens* A136	pCF218-pMV26	luxCDABE reporter (Luminescence-based assay)	C4HSL C6HSL C8HSL C10HSL C12HSL 3OC6HSL 3OC8HSL 3OC10HSL 3OC12HSL	C4HSL (25 nM) C6HSL (250 nM) C8HSL (0.25 nM) C10HSL (25 nM) C12HSL (250 nM) 3OC6HSL (20 pM) 3OC8HSL (0.2 pM) 3OC10HSL (0.02 pM) 3OC12HSL (0.02 pM) [79]
*A. tumefaciens* A136	pCF218-pCF372	LacZ reporter/β-gal	C6HSL 3OC6HSL 3OC8HSL	C6HSL (2.5 µM) 3OC6HSL (100 nM) 3OC8HSL (25 nM) [78]
*A. tumefaciens* KYC55	pJZ384, pJZ410, and pJZ372	LacZ reporter/β-gal	3OC6HSL 3OC8HSL 3OC12HSL 3OHC6HSL 3OHC8HSL 3OHC10HSL C6HSL C8HSL C10HSL	3OC6HSL (2.5 pM) 3OC8HSL (0.25 pM) 3OC12HSL (0.5 nM) 3hydroxylC6HSL (20 pM) 3hydroxylC8HSL (20 pM) 3hydroxylC10HSL (20 pM) C6HSL (100 pM) C8HSL (30 pM) C10HSL (40 pM) [85]
*E. coli* JM109	psB1075	luxCDABE/bioluminescent	C12HSL C14HSL C16HSL 3OC12HSL 3OC14HSL 3OC16HSL 3OHC12HSL	C12HSL (1 nM–50 µM) C14HSL (10 nM–50 µM) C16HSL (100 nM–5 µM) 3OC12HSL (1 nM–5 µM) 3OC14HSL (10 nM–50 µM) 3OC16HSL (10 nM–10 µM) 3OHC12HSL(10 µM–50 µM) [89]
E. coli K-12-Z1	pSB1A2	traI–luxCDABE	Synthetic HSL	1µM [84]
*E. coli*	pUCGMA2T	mCherry	3OC6HSL	5 × 10^−8^–1 × 10^−5^ mol/L [90]
*E. coli* MT102	pJBA132	gfpmut3	C4HSL C6HSL C8HSL 3OC6HSL 3OC10HSL	C4HSL (1 µM) C6HSL (10 nM) C8HSL (10 nM) 3OC6HSL (1 nM) 30C10HSL (10 nM) [91]
*E. coli* DH5α-T1	pSDB1075	LacZ reporter/β-gal w/X-gal immobilized on filter paper	C12HSL	10 nM @ 90 min. 100 nM @ 60 min. [92]
*P. aeruginosa* PA14	pUCP18 and pMS402	luxiCDABE reporter	C4HSL 3OC12HSL	C4HSL (10 µM) 3OC12HSL (10 pM) [86]
*P. aeruginosa* M71LZ	pUCP19	LacZ reporter/β-gal	C4HSL 3OC12HSL	C4HSL (1.0–100 µM) 3OC12HSL (0.01–100 µM) [94]
*P. fluorescens* 1855	pSF105 and pSF107	LacZ reporter/β-gal	3OC6HSL	10–10,000 nM [96]
*P. aeruginosa* PAO1-JP2	pKRC12	GFP	3OC12HSL	25 nM [97]
*P. aeruginosa* PAO1-JP2	pASC8	GFP	C8HSL	25 nM [97]
monkey kidney COS-1	LasBOX 1 sequence	Luciferase	C4HSL 3OC12HSL	Not reported [99]
*A. tumefaciens* NTL4 (cell-free)	pCF218 and pCF372	β-gal	C6 HSL C7HSL C8HSL C10HSL C12HSL 3OC6HSL 3OC8HSL	C6 HSL (30 nM) C7HSL (25 nM) C8HSL (20 nM) C10HSL (100 nM) C12HSL (200 nM) 3OC6HSL (17 nM) 3OC8HSL (10 nM) [101]
*E. coli* extracts (cell-free)	pSB1A2	GFP	3OC12HSL	5–100 nM [103]
*C. violaceum* CV026	Sequenced on genome	violacein	C4HSL C6HSL C8HSL 3OC4HSL 3OC6HSL 3OC12HSL	C4HSL (1.8 nM) C6HSL (0.01 nM) C8HSL (0.44 nM) 3OC4HSL (73 nM) 3OC6HSL (0.14 nM) 3OC12HSL (0.83 nM) [105]
None	3OC12HSL and C4HSL aptamers	electrochemically w/Methylene blue	C4HSL 3OC12HSL	0.1–100 µM [107]
None	*A. tumefaciens* NTL4 reporter	β-gal and PAPG/PAP electrochemical detection	3OC12HSL	2.5 pM (2 h) 3.6 pM (5 h) [108]
